# Geographic Disparities in Evidence Investigating the Use of Biologics in Chronic Rhinosinusitis

**DOI:** 10.1177/19160216261416369

**Published:** 2026-02-14

**Authors:** Itai Margulis, Mohd Afiq Mohd Slim, Ethan C. Sommer, Tatiana Haidar, Zahra Abdallah, Yousif AlAmmar, Sarah Khalife, Doron D. Sommer

**Affiliations:** 1 Division of Otolaryngology—Head & Neck Surgery, Department of Surgery, McMaster University, Hamilton, ON, Canada; 2 Department of Otolaryngology, Forth Valley Royal Hospital, Fakirk, UK; 3 School of Medicine, Dentistry & Nursing, University of Glasgow, Glasgow, UK; 4 Bachelor of Health Sciences Program, Wilfrid Laurier University, Waterloo, ON, Canada; 5 Faculty of Science, Dalhousie University, Halifax, NS, Canada; 6 Department of Otolaryngology—Head & Neck Surgery, King Saud University, Riyadh, Saudi Arabia

**Keywords:** adult rhinology, quality of life, epidemiological studies, immunology, outcomes/cost effectiveness, statistics

## Abstract

**Importance:**

Despite a surge in the volume of evidence assessing the safety and efficacy of biologics for the treatment of chronic rhinosinusitis (CRS), nuances relating to geographic variations in this literature remain insufficiently elucidated.

**Objective:**

To assess the diversity and representation of populations within the literature investigating the use of biological agents for CRS.

**Design/Setting:**

Systematic review.

**Participants:**

Adults ≥18 years with CRS treated with biologic agents.

**Interventions:**

Following PRISMA guidelines, 2 complementary analyses of all studies published between 2006 and 2023 (analysis A), and randomized controlled trials (RCT) and real-world (RW) studies published between 2006 and 2025 (analysis B) were performed.

**Main outcomes measures:**

Patients’ number and country of origin, race/ethnicity, authors’ affiliated countries and Human Development Index (HDI). Types of biologics agents and metrics of the publications were collected.

**Results:**

Out of 2768 studies reviewed, 169 were included in the final analyses. Dupilumab was the most studied biologic agent (37.8%), followed by mepolizumab. The United States had the highest absolute representation and Belgium the highest number of authors per capita, which was correlated with patients’ nationality. The majority of the journals’ and authors’ country of origin was the United States. Only 19 (11.2%) studies disclosed patients’ race/ethnicity, with Asian and Caucasian subjects most commonly represented. The authors’ HDI correlated with journals’ H-index and impact factor. Sixty-seven studies (39.6%) had industry funding, with dupilumab representing the highest number (15.9%).

**Conclusion:**

Although the use of biologics has shown promising results in the management of CRS, most of the evidence comes from the United States and Europe. There is a paucity of representation from certain regions, including Africa, Latin America, and Asia, and inadequate overall disclosure of race/ethnicity in existing studies. This warrants further high-quality investigation of biological agents’ safety and efficacy among these underrepresented populations.

**Relevance:**

Addressing gaps in clinical studies is important for furthering understanding of the pathophysiology and pharmacology of biologic agents for CRS, and bridging treatment disparities.

## Graphical abstract

**Graphical Abstract:**
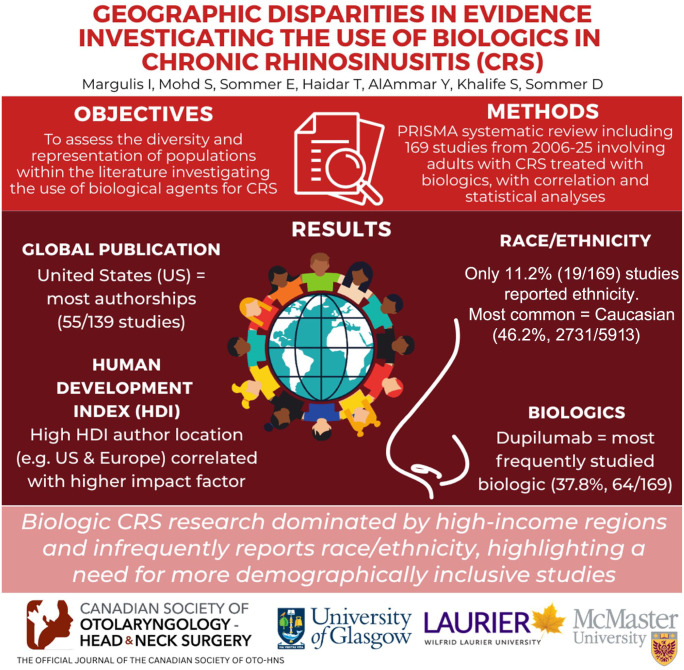

## Key Messages

- Most of the evidence on biologic agents for CRS comes from the United States and Europe, indicating a relative paucity of representation from certain regions.
- Inadequate overall representation and disclosure of race and ethnicity in existing studies warrants further investigation of biological agents’ efficacy on diverse population groups.
- Future studies should aim to increase geographic and ethnic diversity in clinical trials.

## Introduction

Chronic rhinosinusitis (CRS) is one of the most common otolaryngologic disorders and the prevalence varies widely between countries with an estimated range of 5% to 12.1% worldwide.^1^ With respect to specific regions, the prevalence of CRS varies within populations. For example, in Canada and the United States the prevalence ranges between 5.2% and 12.1%, whereas in Asian countries this ranges widely between 2.1% and 28.4%.^1-3^ Our understanding of CRS pathophysiology and current treatment options has increased tremendously over the last 2 decades; however, major gaps remain. Uncertainties persist about differences in CRS pathophysiology and treatment responses across regions. The treatment pathways for CRS have been somewhat based on endotype features with the main treatment options including the following: intranasal and systemic corticosteroids, antibiotics, endoscopic sinus surgery, and biologic agents.^1^

The effectiveness and safety of dupilumab, omalizumab, and mepolizumab in the treatment of CRS with nasal polyps (CRSwNP) were more recently reported in several large real-world studies, independent of the number of sinus surgery procedures, demographic parameters, and comorbidities.^4,5^ Despite this, specifics regarding geographic variations in this literature have not been well addressed thus far. A systematic review by Whitehead et al^6^ concluded that most literature on CRS disparities describes the influence of socioeconomic status and race on disease presentation and progression, and only 15.4% of included studies discussed geographic region as a source of disparity in CRS. Among the parameters used to compare countries’ levels of infrastructure and development is the Human Development Index (HDI), which provides a composite measure of average achievements across key dimensions of human development: longevity/health measures, education, and standard of living.^7^

This review aimed to assess the diversity and representation of populations within the current literature investigating the use of biological agents for CRS.

## Methods

A systematic review adhering to the PRISMA guidelines was conducted.^8^ The PubMed and Web of Science search engines were used to identify publications on the topic of biologics in CRS for adults (≥18-years-old), published in English until August 31, 2025. The study comprised 2 complementary analyses of related datasets. Analysis A included the following study designs from May 2006 to October 2023: case reports, case series, cohort studies, real world (RW), and randomized controlled trials (RCT). Analysis B included all RW and RCTs from May 2006 to August 2025.

Publications encompassing meta-analysis, systematic reviews, position papers, guidelines, gray literature, and unpublished studies were excluded. Cross-referencing was also performed. Patient populations based on CRSwNP were all included. Studies including cohorts of patients with asthma and CRSwNP were included, and the data from the nasal polyps subgroups were extracted.

### Study Selection and Data Collection

Titles and abstracts were screened independently using Covidence^9^ to ensure all studies were included. Any disagreements were resolved by discussion, and if consensus was not met, this was resolved by the senior author (D.D.S.). Data were then extracted by 3 authors (T.H., Z.A., and E.S.) and validated separately by 3 authors (M.A.M.S., Y.A., and I.M.).

### Outcomes

(i) Study data included number and types of publications per year, types of biologic agents, and metrics of the studies’ journals: Journal’s host country, H-Index (HI),^10^ 2023 (analysis A) and 2025 (analysis B) impact factor (IF),^11^ open access availability, industry funding declaration. The HI expresses the journal’s number of articles that have received at least H citations. It quantifies both journal scientific output and impact.^12^
(ii) Patient data included participants number, country of origin, race/ethnicity disclosed by studies. Data on countries population updated to the year of analyses were extracted from the World Development Indicators database.^7^
(iii) Author data included the authors’ number, authors’ countries of origin, HDI, and gross domestic product (GDP) extracted from online databases.^13,14^ The most recent HDI values were used up to the time of analysis A (2021) and B (2023) and similarly for GDP (2022 and 2023, respectively).

### Data Analysis

Descriptive analysis of the data was conducted using the Jamovi software.^15^ Figures and mapping data representation were performed using Microsoft Excel. Associations between the categorical covariates of interest were explored using the chi-squared test (*X*^2^) and Fisher’s exact (*f*) test and expressed in odds ratio (OR). The Shapiro-Wilk test was performed to evaluate normality of variables. Pearson correlation was used for normal variables and Spearman rank correlation (rho) for non-normal variables, specifically the journal’s IF and HI, the authors country’s HFI and GDP. Significance was set at *P* ≤ 0.05.

## Results

**Figure 1.**
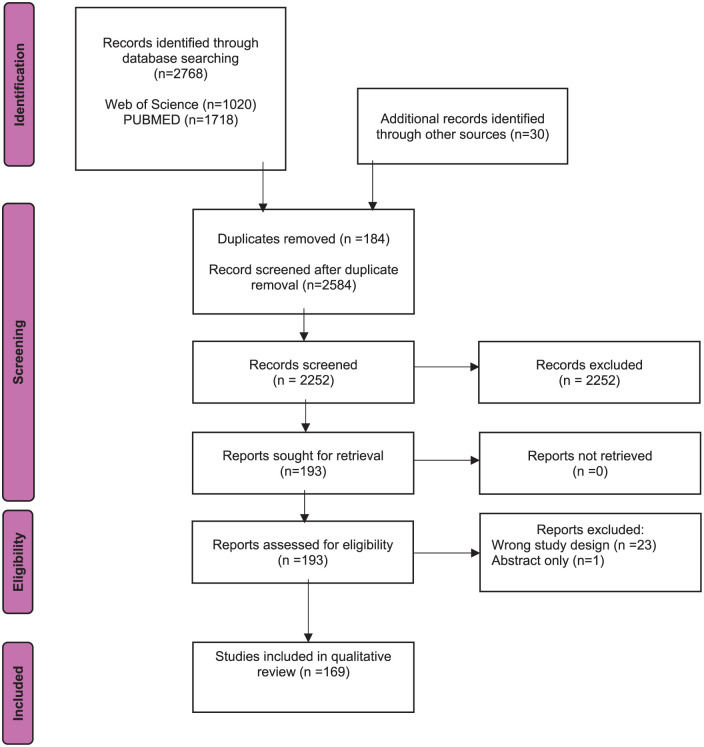
PRISMA flow chart detailing search strategy and reasons for exclusion. PRISMA, Preferred reporting items for systematic reviews and meta-analyses.

A total of 2768 publications were identified following the search with 169 publications selected for this review (Figure 1). Analysis A included 139 studies, and analysis B included 76 studies.

### Studies Characteristics

#### Analysis A (2006-2023)

**Figure 2.**
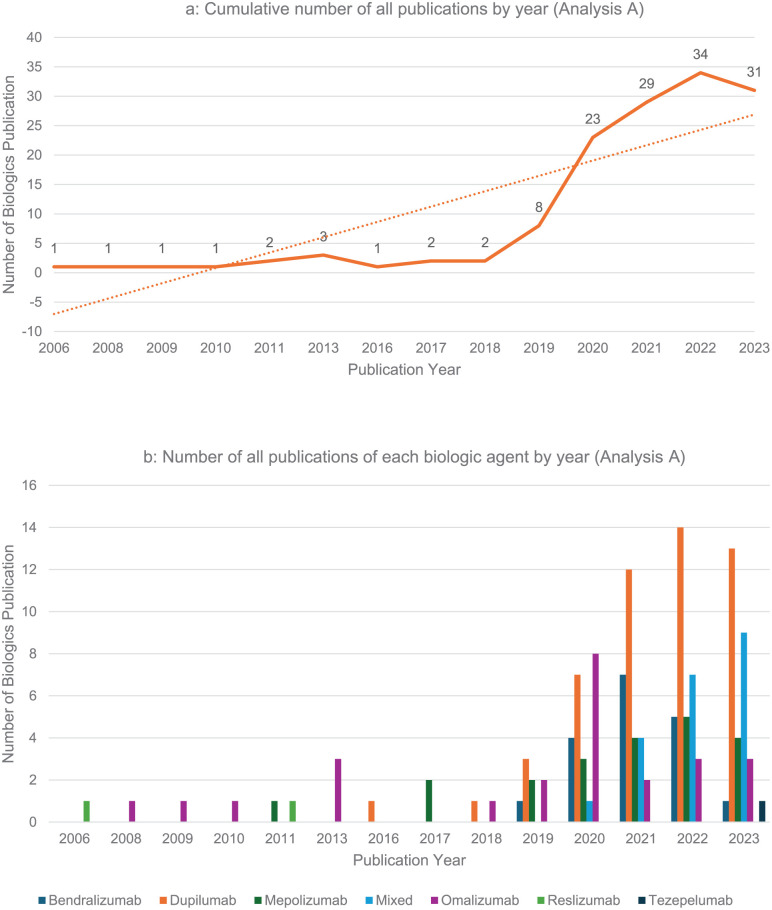
Publication trends by year for biologics in CRS in analysis A. The figures present a line graph with an increasing number of all biologics publications and of each biologic agent by publication year 2006 to 2023. (a) Cumulative number of all publications by year (analysis A); (b) Number of all publications of each biologic agent by year (analysis A). CRS, chronic rhinosinusitis.

- Publication trends: Among the 139 included studies an increasing trend in the number of publications of biologics for CRS can be seen since 2006, with a rapid rise since 2018 and a tripling in the number of publications around 2020 (Figure 2a). RCTs represented the most common type of study design within the studies composing 27.8% of studies (n = 47, Table 1). Dupilumab was the most studied biologic accounting for 51 (36.7%) of the studies (Figure 2b, Table S2).
- Journals characteristics: The median HI of the host journal was 67 [50; 159], and the median journal’s IF was 5.10 [2.60; 7.20]. The most common journal country of origin was the United States (n = 55, 39.9%). Overall, 54/139 (38.8%) studies were funded by pharmaceutical companies. Dupilumab, as a single or part of multiple studied agents, had the highest number of funded studies (n = 63, 45.3%), and mepolizumab was the second most funded (n = 36, 25.9%). Open access was available for 78.4% (n = 109) with industry funding support encompassing 38.8% (n = 54) of the current publications.

**Table 1.** Descriptive Summary of all Studies Included.

Studys’ characteristics	Overall no. (%) (N = 169)
Type of study	
Randomized control trials	47 (27.8)
Cohort study	46 (27.2)
Case report	32 (18.9)
Real-world studies	30 (17.7)
Case series	12 (7.1)
Case control	1 (0.6)
Case matched	1 (0.6)
Type of biologics	
Dupilumab	64 (37.8)
Omalizumab	27 (15.9)
Mepolizumab	28 (16.5)
Mixed	25 (14.7)
Benralizumab	21 (12.4)
Tezepelumab	2 (1.2)
Reslizumab	2 (1.2)
Depemokimab	1 (0.6)
Funding from pharmaceutical company	
Yes	67 (39.6)
No	102 (60.3)
Access available without subscription	
Yes	125 (73.9)
No	44 (26)
Subjects’ race and ethnicity disclosure—add race according to countries	
Yes	19 (11.2)
No	150 (88.7)

#### Analysis B (2006-2025)

**Figure 3.**
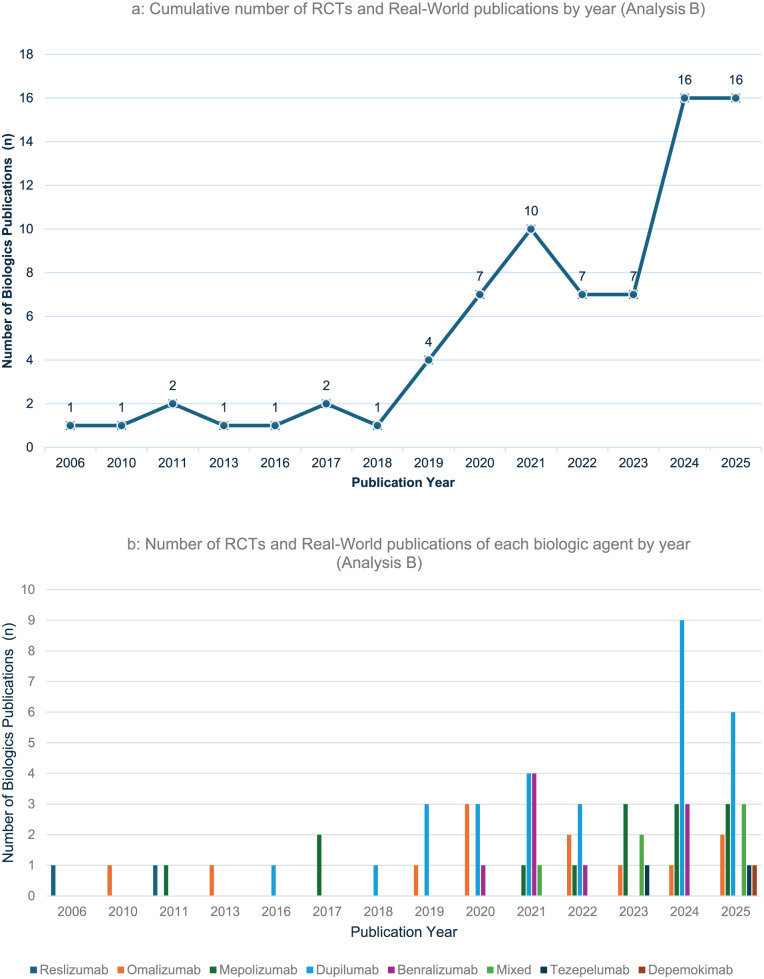
Publication trends by year for biologics in CRS in analysis B. The figures present a line graph with an increasing number of RCTs and real-world biologics publications and of each biologic agent by publication year 2006 to 2025. Cumulative number of RCTs and real-world publications by year (analysis B) (b) Number of RCTs and real-world publications of each biologic agent by year (analysis B). CRS, chronic rhinosinusitis; RCT, randomized controlled trial.

- Publication trends: Among the 76 RCT and RW studies included, the number of these study types has been sharply increasing since 2019, roughly doubling yearly during 2019 to 2021 and 2024 (Figure 3a). Dupilumab was the most studied biologic in 30 (39%) of studies, followed by mepolizumab (14 studies, 18%) (Figure 3b, Table S2).
- Journal characteristics: The median 2025 HI of the host journal was 60 [37.5; 97], and the 2025 IF was 6.8 [3.0; 11.2]. Most of the journals’ host country of origin was the United States (n = 36, 47%). Open access was available by 70% (n = 53) with industry funding support encompassing 64% (n = 49) of the current publications (Supplemental Table S3). Overall, 49/76 (64.4%) studies were funded by pharmaceutical companies. Dupilumab, as a single or part of multiple studied agents, had the highest number of funded studies (n = 21/46, 42.8%), followed by mepolizumab (n = 11/46, 23.9%).

### Authors’ Characteristics

**Figure 4.**
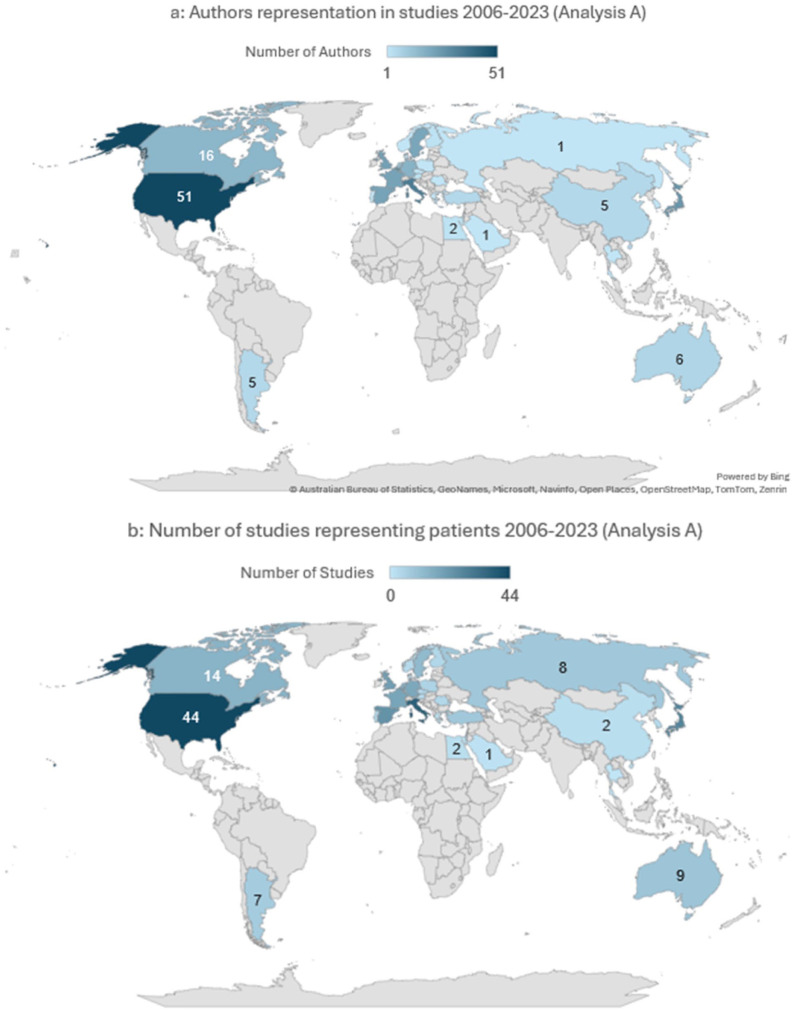
Geographic distribution of authors and patients in all studies published between 2006 and 2023 in analysis A. (a) Total number of authors represented by country. This world map illustrates the number of contributing authors in each country. Countries are color-coded based on the total number of authors, with darker shades indicating higher author representation. Countries with no affiliated authors are shown in gray. (b) Total number of studies represented by country. This world map shows the number of studies recruiting patients in each country. Countries are color-coded according to the total number of studies, with darker shades representing a higher number of studies.

**Figure 5.**
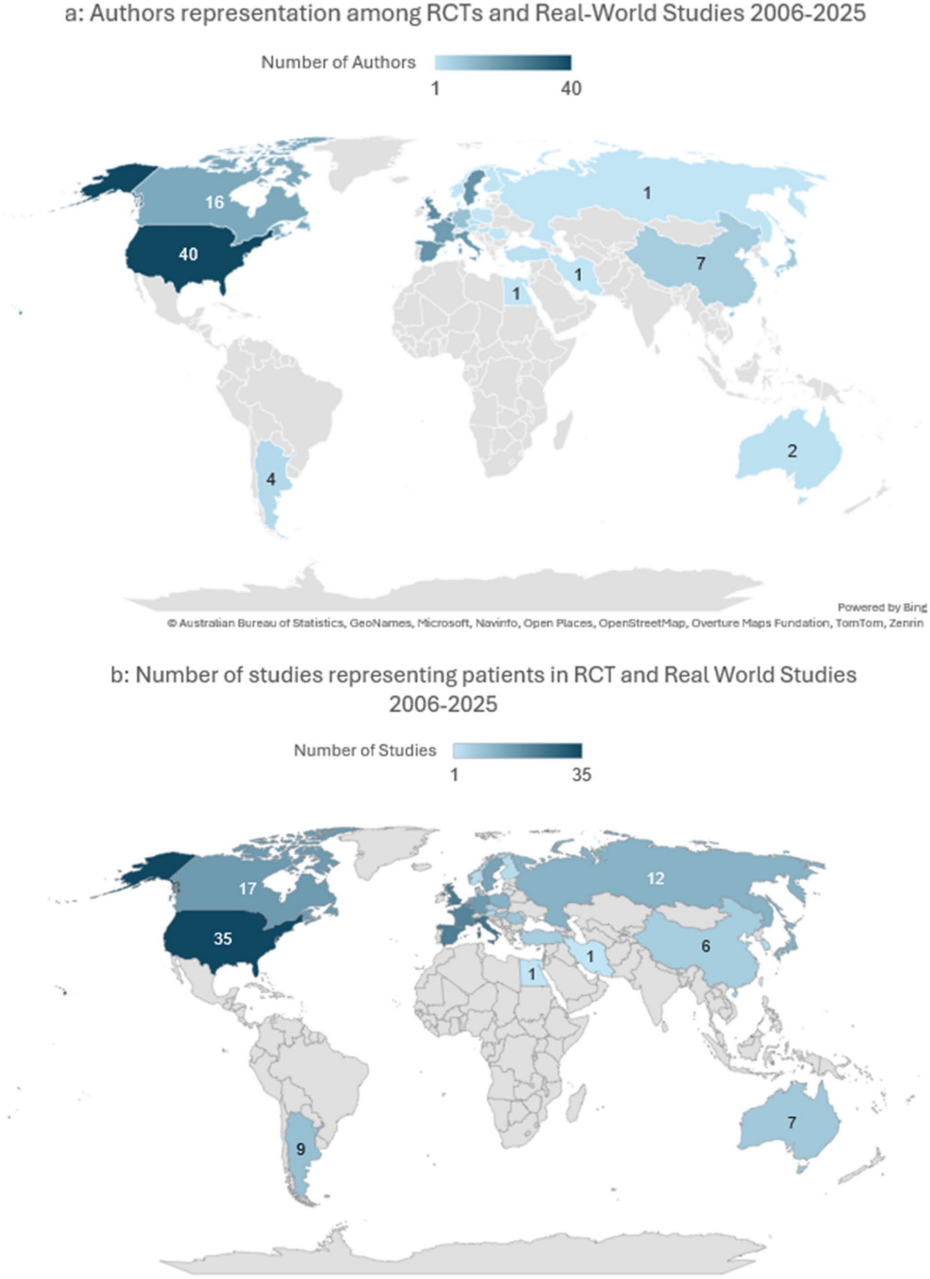
Geographic distribution of authors and patients in RCTs and real-world studies published between 2006 and 2025 in analysis B. (a) Total number of authors represented by country. This world map illustrates the number of contributing authors in each country. Countries are color-coded based on the total number of authors, with darker shades indicating higher author representation. Countries with no affiliated authors are shown in gray. (b) Total number of studies represented by country. This world map shows the number of studies recruiting patients in each country. Countries are color-coded according to the total number of studies, with darker shades representing a higher number of studies. RCT, randomized controlled trial.

- Geographic author distribution in analysis A: Overall, 29 countries were represented by authorship (n = 314, Table 2a) with the United States having the highest absolute authors representation (n = 51, 16.2%) (Figure 4a) and Belgium the highest per capita (2.40 per million population, Table 2a). Notably, the country with the highest number of first authors was Italy (n = 29, 20.9%, Table S1). No significant differences were seen based on the authors’ continent (*P* = .426). The median HDI average of the authors was 0.921 [0.895; 0.925]. Strong correlation was noted between the authors’ country of origin and the number of participating countries of origin (rho: 0.717, *P* < .001).
- Geographic author distribution in analysis B: Overall, 28 countries were represented by named authorship (n = 257, Table 2b, not including large group publications secondary authors, eg, SYNAPSE group), with the United States having the most absolute representation (n = 40, 15.5%) (Figure 5a) and Belgium representing the highest per capita (2.74 per million population, Table 2b). Notably, the country with the highest number of first authors was Belgium (n = 17, 22%, Table S1). There was a significant difference between the authors’ distribution based on continent (X^2^: 540, *P* < .001) with those from Europe being the highest 68%. The median 2023 HDI of the authors was 0.931 [0.915; 0.941].

**Table 2a.** Overall Representation of Authors and Patients by Studies in Analysis A (2006-2023).

Country	Number of authors representation (n)	Per 1 mil authors representation	Number of studies representing patients*	Per 1 mil population represented by the studies
Argentina	5	0.11	7	0.151
Australia	6	0.23	9	0.346
Austria	2	0.22	3	0.332
Belgium	28	2.40	20	1.714
Brazil	n/a	0.00	1	0.005
Bulgaria	n/a	0.00	4	0.619
Canada	16	0.41	14	0.360
China	5	0.00	2	0.001
Czech Republic	2	0.19	4	0.380
Colombia	n/a	0.00	1	0.019
Denmark	1	0.17	2	0.339
Egypt	2	0.02	2	0.018
Estonia	n/a	0.00	1	0.743
Finland	2	0.36	4	0.720
France	22	0.32	18	0.265
Germany	17	0.20	17	0.202
Greece	1	0.09	2	0.189
Israel	2	0.21	5	0.524
Italy	34	0.58	37	0.629
Japan	26	0.21	27	0.216
South Korea	1	0.02	3	0.058
The Netherlands	13	0.73	15	0.853
Norway	1	0.18	3	0.550
Peru	n/a	0.00	1	0.029
Poland	1	0.03	5	0.133
Portugal	1	0.10	4	0.385
Romania	1	0.05	4	0.211
Russia	1	0.01	8	0.056
Saudi Arabia	n/a	0.00	1	0.027
Slovakia	n/a	0.00	1	0.1841
South Africa	n/a	0.00	1	0.017
Spain	22	0.46	23	0.483
Sweden	21	2.00	12	1.144
Thailand	1	0.01	n/a	0.000
Taiwan	n/a	0.00	1	0.042
Turkiye	5	0.06	8	0.094
The United Kingdom	23	0.34	20	0.299
The United States	51	0.15	44	0.132

Abbreviations: mil, Million; n/a, not available- the country had no author/s in the included studies.

* The number refers to the number of studies recruiting patients in the specific country, including multicentric studies.

**Table 2b.** Overall Representation of Authors and Patients by Studies in Analysis B (2006-2025).

Country	Number of authors representation (n)	Per 1 mil authors representation	Number of studies representing patients *	Per 1 mil population represented by the studies
Argentina	4	0.087	9	0.195
Australia	2	0.077	7	0.269
Austria	3	0.332	4	0.442
Belgium	32	2.742	20	1.714
Canada	16	0.411	17	0.437
Chile	n/a	0.000	4	0.205
China	7	0.005	6	0.004
Czech Republic	2	0.190	5	0.475
Denmark	2	0.339	4	0.678
Egypt	1	0.009	1	0.009
Estonia	n/a	0.000	1	0.751
Finland	1	0.180	3	0.540
France	19	0.280	22	0.325
Germany	10	0.119	17	0.202
Hungary	1	0.103	8	0.826
Iran	1	0.011	1	0.011
Israel	1	0.105	5	0.524
Italy	20	0.340	25	0.425
Japan	8	0.064	12	0.096
Mexico	n/a	0.000	6	0.047
The Netherlands	14	0.780	15	0.836
Norway	1	0.183	3	0.550
Peru	n/a	0.000	1	0.029
Portugal	n/a	0.000	5	0.482
Poland	1	0.027	12	0.319
Romania	1	0.053	8	0.422
Russia	1	0.007	12	0.084
South Korea	2	0.039	3	0.058
Saudi Arabia	n/a	0.000	1	0.029
Slovakia	n/a	0.000	1	0.183
Spain	21	0.441	23	0.483
Sweden	21	2.002	15	1.430
Ukraine	n/a	0.000	7	0.171
Turkey	2	0.023	6	0.070
The United Kingdom	23	0.343	24	0.358
The United States	40	0.120	35	0.105

Abbreviations: mil, million; n/a, not available—the country had no author/s in the included studies.

* The number refers to the number of studies recruiting patients in the specific country, including multicentric studies.

### Patients’ Characteristics

- Patients’ representation in analysis A: Of 139 studies, the median participants’ sample size was 34.5 [3; 126], the median number of males was 18.5 [3.75; 84.0] and of female was 20.5 [5.0; 78.3]. Patients’ nationality was represented by 38 countries, with the United States having the largest contribution (n = 44, Table 2a) (Figure 4b), and Belgium having the highest concentration at 1.71 participation in the study per million population (Table 2a). There were significant differences in representation based on the patients’ continent (*P* = .023) whereby those from Europe contributed the highest representation (*P* = .016).
- Patients’ representation in analysis B: Of 76 studies, the median participants’ sample size was 143 [56.8; 407], the median number of males was 84 [25.5; 257] and of female was 62.0 [5.0; 143]. Patients’ nationality was represented by 37 countries, with the United States having the largest proportion (n = 35 studies, Table 2b) and Belgium having the highest concentration at 1.714 participation in the study per million population (Table 2b). There was a significant difference between the patients’ sampling distribution based on the continent (X^2^: 714, *P* < .001) with Europe being the highest 68.1%. There was a strong correlation between the patients’ continent and the authors’ continent (rho: 1, *P* = .003).
- Race/Ethnicity disclosure in all included studies: Overall, only 19/169 (11.2%) studies (within analyses A and B) disclosed their patients’ race and ethnicity, as summarized in Table 3. Among 5913 participants in those studies, Caucasian was the most common (n = 2731, 46.2%), followed by Asians from all regions (n = 1061, 17.9%). The least represented ethnicities were Hispanic/Latino (n = 24), Native Hawaiian/other pacific islander (n = 1), Arabic/North African (n = 1), and Native American (n = 1).

**Table 3.** Overall Representation of Race and Ethnicity of Participants Disclosed by 19 Studies.

Study	Number of participants *	White, n	Non-whites, n	Black and African American, n	Asians (all regions), n	Arabic and North African	Native American	Native Hawaiian or other pacific islander	Other or multiple	Hispanic or Latino, n	Non Hispanic or Latino, n	Missing **
Persaud et al. 2022	1	1	n/a	n/a	n/a	n/a	n/a	n/a	n/a	n/a	n/a	n/a
Lombardo et al. 2024	40	34	6	2	n/a	n/a	n/a	n/a	1	3	n/a	n/a
Garvey et al. 2023	32	18	14	n/a	n/a	n/a	7	n/a	n/a	7	n/a	n/a
D’Souza et al. 2021	46	32	14	n/a	n/a	n/a	n/a	n/a	n/a	n/a	n/a	n/a
Bachert et al. 2016	60	59	1	n/a	n/a	n/a	n/a	n/a	n/a	n/a	n/a	n/a
Mullur et al. 2025	98	88	10	2	98	1	n/a	n/a	5	n/a	n/a	3
Bachert et al. 2017	105	102	3	n/a	108	n/a	n/a	n/a	n/a	n/a	n/a	n/a
Dharmarajan et al. 2021	108	93	15	11	n/a	n/a	n/a	n/a	n/a	n/a	n/a	4
Canonica et al. 2021	153	114	39	n/a	8	n/a	n/a	n/a	3	n/a	n/a	n/a
Fujieda et al. 2024	163	48	115	n/a	115	n/a	n/a	n/a	n/a	n/a	n/a	n/a
Patel et al. 2021	165	107	58	32	10	n/a	n/a	n/a	16	n/a	149	n/a
Emson et al. 2024	185	178	7	5	186	n/a	n/a	n/a	1	n/a	n/a	n/a
Gore et al. 2022	241	178	n/a	44	4	n/a	1	n/a	14	14	n/a	n/a
Damask et al. 2022	265	257	8	n/a	2	n/a	n/a	n/a	2	n/a	n/a	n/a
Han et al. 2021	407	373	34	53	381	6	n/a	n/a	1	n/a	n/a	n/a
Gevaert et al. 2025	528	380	148	n/a	n/a	n/a	n/a	n/a	n/a	n/a	n/a	n/a
Harrison et al. 2021	656	482	174	74	71	n/a	n/a	1	7	n/a	n/a	n/a
Berger et al. 2023	378	178	47	5	39	n/a	n/a	n/a	1	n/a	n/a	n/a
Jonstam et al. 2018	2282	59	1	n/a	n/a	n/a	n/a	n/a	n/a	n/a	n/a	n/a

Abbreviations: “n/a,” not available—no representation was reported in the included study.

* The total number of patients with disclosed race and ethnicity is less than the overall study population due to missing data.

** The missing number of participants was documented based on the studies’ report and not on calculation.

### The Effect of Authors’ HDI on Studies and Patients’ Characteristics

#### Analysis A

Significant correlation was found between the authors’ average 2021 HDI, the host journal’s 2022 HI (rho: 0.220, *P* = .01) and 2023 IF (rho: 0.202, *P* = .019). Conversely, no correlation was found between the authors’ average HDI and the number of Caucasian (rho: −0.075, *P* = .746), non-Caucasian (rho: −0.053, *P* = .818), male (rho: 0.008, *P* = .930), and female (rho: −0.056, *P* = .536) patients represented in the studies.

In addition, no associations were noted between the average authors’ HDI and the type of biologic publications (*P* = .713). This is true for both patients’ race (OR = 0.550, 95% CI: 0.081-3.70, *P* = .319) and ethnicity (OR = 0.488, 95% CI: 0.042-5.63, *P* = .289) disclosures. The average authors’ HDI did not show a correlation with industry funding (OR = 0.957, 95% CI: 0.561-1.63, *P* = .957) or publication open access status (OR = 0.890, 95% CI: 0.515-1.54, *P* = .678).

#### Analysis B

The type of biologic agents represented in the RW and RCT studies was correlated with the average author’s 2023 HDI only for mepolizumab (*P* = .03), as single or as part of multiple studied agents, but not for all other biologic agents. Interestingly, significant correlation was found between the authors’ average 2025 HDI, the host journal’s 2025 HI (rho: 0.298, *P* = .012), and the host journal’s 2025 IF (rho: 0.476, *P* < .001).

The average authors’ HDI did not show a correlation with race (*P* = .86) or ethnicity (*P* = .2) disclosure nor with industry funding supplementation (OR = 6.437, 95% CI: 0.009-4347, *P* = .575) or publications’ open access status (OR = 4.662, 95% CI: 0.005-3831, *P* = .653).

No correlation was seen between the authors’ average HDI and the number of Caucasian (*P* = .22), non-Caucasian (*P* = .53), male (*P* = .38), and female (*P* = .52) patients represented in the studies.

### The Effect of Industry Funding on Studies and Patients’ Characteristics

#### Analysis A

Industry funding supplementation varied significantly (*P* = .001) based on the type of biologic agent studies, with mepolizumab showing the greatest association with funding (OR = 22.0, 95% CI: 2.479-195.267, *P* = .006, Table S3). Reslizumab and tezepelumab were excluded in industry funding analysis due to inadequate sample size. The majority of the studies receiving industry support also disclosed their patients’ race (OR = 9.052, 95% CI: 3.358-24.403, *P* < .001) and ethnicity (OR = 16.2, 95% CI: 5.189-50.571, *P* < .001); however, the sample size of studies disclosing race/ethnicity was small (7/139 studies, 5%).

Statistically-significant correlation was also found between industry funding support and the host journal’s 2022 HI (OR = 1.00, 95% CI: 1.005-1.014, *P* < .01) and 2023 IF (OR = 1.190, 95% CI: 1.086-1.304, *P* < .01). An association is also evident between industry funding and the proportion of Caucasian (OR = 1.015, 95% CI: 11-1.03, *P* < .01) patients recruited into the study. No correlation was seen for non-Caucasian (OR = 1.01, 95% CI: 0.987-1.03, *P* = .276) patients. Interestingly, open access publication status was not associated with funding (OR = 1.125, 95% CI: 0.488-2.60, *P* = .782).

#### Analysis B

Industry funding varied significantly (*P* = .042) based on the type of biologic publications available, with mepolizumab showing the greatest association with studies’ funding (OR = 18.3, 95% CI: 1.5-222.9, *P* = .022), followed by dupilumab OR = 11.7, 95% CI: 1.18-114.6, *P* = .03) (Table S3). The majority of the studies receiving industry funding support also disclosed their patients’ race (OR = 8.67, 95% CI: 1.06-70.9, *P* = .044) but not their ethnicity (*P* = .13); however, the sample size of studies disclosing race/ethnicity was small (13/76 studies, 17.1%). Funding support also correlated with the host journal’s 2025 IF (OR = 1.3, 95% CI: 1.11-1.55, *P* = .002). However, industry funding was not associated with open access publication status (*P* = .34) and journal’s 2025 HI (*P* = .12).

### The Effects of Biologics Agent Types on Studies and Patients’ Characteristics

#### Analysis A

No significant correlations were found between the various types of biologic agents used, patients’ race (*P* = .386), ethnicity (*P* = .241), or author’s 2022 GDP (*P* = .6). The host journal country’s 2022 HI (*P* = .078), 2023 IF (*P* = .442), and open access status (*P* = .359) also did not yield any significant differences.

#### Analysis B

All biologic agents studied were correlated with patients’ race (*P* < .001) and ethnicity (*P* < .001) disclosure. The only biologic agents that correlated with journal’s 2025 IF was depemokimab (OR = 3.0, 95% CI: 2.32-3.88, *P* < .001). Only tezepelumab was correlated with journal 2025 HI (OR = 1.01, 95% CI = 1.0-1.03, *P* = .04)

However, biologic agent types were not associated with the average authors’ 2023 GDP (*P* = 1.0) and open access status (*P* = .4).

## Discussion

To the best of our knowledge, this is the first study to specifically investigate geographic and demographic disparities in biologics research for CRS.

Our study shows that geographic representation is mainly concentrated in the United States and Europe, while outside of these regions, Japan is well represented. On the other hand, there was little to no representation from certain regions, such as Africa, Southeast Asia, and South America. In addition, there is a paucity of data published regarding the correlation between biologic agents use for CRS and patients’ race and ethnicity.

### Correlation Between Biological Agents, Authors’ HDI and Industry Funding

Over the study period, there was a notable increase in the number of articles published about biologic agents for CRS with dupilumab being the most studied (37.8%) agent followed by mepolizumab (16.5%). Dupilumab had the highest number of industry-funded studies.

Interestingly, we found no significant differences or associations between the average authors’ HDI and the type of biologic agent used, except for mepolizumab in analysis B. This may be explained by other influencing factors, such as the availability of biologic agents in different countries, common practice guidelines implementation for CRS, industry funding and patients’ insurance status that effect choosing a biologic agent.

However, the average HDI was correlated with the host journal country’s HI and IF with a significant proportion of these journals published in the United States (39.5% and 47.3% in analyses A and B, respectively).

This lack of representation of low-HDI countries has been demonstrated across other fields of medicine, such as cardiothoracic surgery, anesthesia, general surgery, ophthalmology, and medical education, within which the majority of publications originated from North America and Western Europe, followed by Japan and China.^16-18^ In a recent bibliometric analysis of the trends in CRS treatment from 2001 to 2020, most of the articles came from relatively-high-HDI countries such as the United States (1390 Articles, 42.9%) followed by China (242 Articles, 7.4%) and the UK (230, 7.1%).^19^

### Race and Ethnicity Disclosure Among Studies

In our review, the majority of studies did not disclose their subjects’ race and ethnicity (111, 79.9%). Among the 19 studies (11.2%) disclosing race and ethnicity, Caucasians were the most represented race, and Asians were the largest reported ethnic group. This proportion of race and ethnicity representation is lower than reported in a large cohort study of US-based clinical trials (45%), while, similarly, the majority of enrollees in this study were Caucasians (81.1%) and Asians (9.7%).^20^

In contrast, when assessing industry-funded trials, the majority of studies receiving industry funding did disclose their patients’ race (in both analyses) and ethnicity (analysis A only). There was an association between industry-funded studies and the number of Caucasian patients recruited into the study (*P* < .01), and this association was not seen for non-Caucasian patients (*P* = .276). On the other hand, no association was seen between the mean authors’ HDI and the race, ethnicity, or gender of the patients recruited in the studies, regardless of the type of biologic agent used.

These findings contrast with those of Turner et al, who analyzed all US clinical trials conducted between 2000 and 2020 and found that both industry and academic funding were negatively associated with the reporting of race and ethnicity. They also reported that industry-funded trials had a lower proportion of participants from minority ethnic groups compared to those funded by the US government.^20^

A possible explanation for why our findings differ from that study is that the overall number of studies in our review that disclosed race and ethnicity was small (19 studies, 11.2%), which may have influenced the observed correlation with industry funding. This relationship might change with larger sample sizes.

The potential use for biological agents in the treatment of CRS crosses all race and ethnic groups, and it remains unclear whether differences exist in their efficacy among different populations. As there may be differences in CRS endotypes and inflammatory patterns across regions, this is an important area for further investigation. Future real-world studies should address disparities in their cohorts for generalizability of biologic agents’ efficacy. The lack of diversity in studies may be a function of the primary location of the majority of the studies (United States and Europe). Industry-funded clinical trials often choose study locations based on infrastructure and the ability to meet strict study specifications and inclusion and exclusion criteria, which in turn leads to decreased diversity. Given the critical role that industry funding plays, a more active role should be taken to bridge this gap by encouraging appropriate geographic diversity. Literature with underrepresentation in race and ethnicity variability raises questions about the generalizability of results for clinical decision-making. Recent publications regarding diversity in medicine have increasingly highlighted the significance of race and ethnicity among studied populations. In a trial of dupilumab in children with uncontrolled asthma, most of the children (90%) were of European origin, forming an imbalance of representation.^21^ The need to address racial and ethnic disparity among clinical trials recruitment and participation was also emphasized by the American Society of Clinical Oncology (ASCO) and the US Food and Drug Administration (US FDA).^22,23^ The ASCO recommended that trial sponsors and investigators should design trials with a focus on reducing barriers and increasing participation of underrepresented populations, as well as detailing data on racial and ethnic diversity of trial participants.^22^ The US FDA issued a draft guidance, which focuses on racial and ethnic diversity while encouraging inclusion of other underrepresented populations relevant to disease areas, including gender identity, age, and socioeconomic status. In addition, the FDA recommends strengthening collaboration among stakeholders will help reduce barriers and enrich diversity and equity in clinical research and reduce disparities and gaps in treatment ultimately providing a positive impact on public health and patient safety.^23^ Loree et al addressed these recommendations when evaluating the frequency of race reporting and representation in trials supporting FDA oncologic drug approvals. Compared with their proportion of US cancer incidence, African American and Hispanic subjects were underrepresented compared with Caucasian and Asian subjects.^24^

Furthermore, concerns may be raised regarding possible differences in pharmacokinetics and pharmacodynamics of biological agents for CRS among different racial groups. In a study of dupilumab on intractable CRS in a Japanese population, no notable pharmacokinetic differences between non-Japanese and Japanese patients were observed, with only a slightly-higher serum concentration of dupilumab likely as a consequence of the tendency toward a lower body weight in the latter group.^25^ Another example is warfarin’s pharmacodynamics differences seen in 3 racial groups (Asian, Caucasian, and African American patients).^26^

### Geographic Variations in CRS

Geographic variations were demonstrated in a study of leading general surgery journals, showing almost complete lack of representation of low- and medium-HDI countries in these journals.^18^ One review on asthma investigated geographic and ethnic disparities between treatment groups, but it did not focus on biologic therapy for asthma.^27^

This association might be related to several factors. First, lower HDI countries may be prioritizing publications assessing more high yield, life-saving, and large-impact interventions (ie, malaria treatment, access to care), as opposed to publications investigating relatively-costly treatments, which mostly impact quality of life. Second, the limited institutional and educational infrastructure in certain regions may be a barrier to conducting large studies with appropriate monitoring and assessments (ie, CT scanning, nasal endoscopy) needed for biologic trials for CRS. As noted, the prevalence of CRS and associated morbidity may not represent as substantial a burden in some regions/countries. ^2^ Furthermore, the predominance of English as the primary language in certain countries may serve as a significant influencing factor in the selection of enrollment centers for CRS studies, potentially resulting in underrepresentation of non-English-speaking regions. Pharmaceutical industry support may not be equally dispersed in such regions, and patients’ and insurance companies’ ability to support these treatments might be low. Finally, there is a somewhat controversial body of evidence within literature assessing geographic variations in CRS. Much of this deals with perceived variations in rates of eosinophilic CRS.^28,29^ Although much of this may be explained by variations in eosinophilic cutoff scores, it nonetheless points to possible differences, which may be attributed to local geographic factors or racial/ethnic differences within CRS.^30^ It would thus be crucial to expand the geographic/racial/ethnic literature of biologic studies for CRS to identify any potential differences in response to the various biologic treatments for CRS. Future studies of biologics for CRS should facilitate the generation and dissemination of research from low and middle HDI countries. In addition, they should highlight associations of race and ethnicity with biologic treatment’s health outcomes, and how race and ethnicity intersects with other sociodemographic factors.

### Limitations

The nature of this systematic review lends itself to publication bias, where favorable studies are more likely to be published. The retrospective nature of some of the included studies carries selection bias. Grey literature, such as conferences abstracts or white papers, was excluded from our review, which could lead to underrepresentation of some populations. However, we believe that the included studies in this review well-represent the majority of studies investigating the use of biological agents for CRS. In addition, this review was comprised of 2 complementary analyses carried out in separate time points (2023 and 2025) and thus using different yearly values of authors’ HDI and GDP and journal’s IF and HI.

The lack of representation of low-HDI countries, as well as disparities in funding and race/ethnicity disclosure, all possibly affect the results of the included studies. Pharmaceutical industry funding may also lead to disparities in geographic site selection and access to biologic agents, depending on regional concerns and industry representation.

Lastly, our review was limited to English language studies and excluded studies in other languages, which could lead to a bias in geographic and ethnic diversity representation. Among the 19 studies that reported participants’ race/ethnicity, some had missing data regarding their participants’ ethnicity (Table 3).

## Conclusion

The use of biologics has shown promising results in the management of CRS. However, a large proportion of the evidence comes from high-HDI countries in the United States and Europe, whereas there is a paucity of representation from Africa, Latin America, and Asia. These warrant focused investigation of biological agents’ efficacy and safety by high-quality studies on diverse population groups in those areas, including low-HDI countries. Race and Ethnicity are not routinely included in the current literature, disclosed only in 11% of studies in our review and potentially limiting our understanding of possible differences in presentations and treatment outcomes. Addressing this knowledge gap is essential for furthering our understanding of the pathophysiology and pharmacology of biologics agents for CRS and bridging treatment disparities. Future studies should address those barriers to increase equity and diversity in trials participation.

## Supplemental Material

sj-docx-1-ohn-10.1177_19160216261416369 – Supplemental material for Geographic Disparities in Evidence Investigating the Use of Biologics in Chronic Rhinosinusitis Supplemental material, sj-docx-1-ohn-10.1177_19160216261416369 for Geographic Disparities in Evidence Investigating the Use of Biologics in Chronic Rhinosinusitis by Itai Margulis, Mohd Afiq Mohd Slim, Ethan C. Sommer, Tatiana Haidar, Zahra Abdallah, Yousif AlAmmar, Sarah Khalife and Doron D. Sommer in Journal of Otolaryngology - Head & Neck Surgery
