# Supplementary material for: Geographic Disparities in Evidence Investigating the Use of Biologics in Chronic Rhinosinusitis
Source: J Otolaryngol Head Neck Surg. 2026 Feb 14;55:19160216261416369. doi: 10.1177/19160216261416369 (PMC12906625; doi:10.1177/19160216261416369)
Supplement: sj-docx-1-ohn-10.1177_19160216261416369 – Supplemental material for Geographic Disparities in Evidence Investigating the Use of Biologics in Chronic Rhinosinusitis [file sj-docx-1-ohn-10.1177_19160216261416369.docx]

**Supplemental material for "Geographic Disparities in Evidence Investigating the Use of Biologics in Chronic Rhinosinusitis"**

Table S1. Overall Country Representation Based on Publication

| **Studys' Characteristics** | **All studies 2006-2023 (Analysis A)**  **N= 139**  **n (%)** | **RCT and RW Studies 2006-2025 (Analysis B)**  **N=76**  **n (%)** |
| --- | --- | --- |
| **First Author's Country of Origin** | | |
| Argentina | 1 (0.7%) | 1 (1.3%) |
| Australia | 3 (2.2%) | n/a |
| Belgium | 14 (10.1%) | 17 (22.4%) |
| Canada | 3 (2.2%) | 3 (3.9%) |
| China | 1 (0.7%) | n/a |
| Denmark | n/a | 1 (1.3%) |
| Egypt | 2 (1.4%) | 1 (1.3%) |
| Finland | 1 (0.7%) | n/a |
| France | 4 (2.9%) | 1 (1.3%) |
| Germany | 6 (4.3%) | n/a |
| Greece | 1 (0.7%) | n/a |
| Hungary | n/a | 1 (1.3%) |
| Israel | 2 (1.4%) | 1 (1.3%) |
| Iran | n/a | 1 (1.3%) |
| Italy | 29 (20.9%) | 14 (18.4%) |
| Japan | 24 (17.3%) | 6 (7.9%) |
| Netherlands | 5 (3.6%) | 4 (5.2%) |
| Portugal | 1 (0.7%) | n/a |
| Poland | n/a | 1 (1.3%) |
| Saudi Arabia | 1 (0.7%) | n/a |
| South Korea | n/a | 1 (1.3%) |
| Spain | 6 (4.3%) | 3 (3.9%) |
| Sweden | 1 (0.7%) | 1 (1.3%) |
| Turkiye | 5 (3.6%) | 1 (1.3%) |
| UK | 4 (2.9%) | 4 (5.2%) |
| USA | 25 (18.0%) | 14 (18.4%) |
| **Host Journal's Country of Origin** | | |
| Egypt | 1 (0.7%) | 1 (1.3%) |
| Europe * | n/a | 1 (1.3%) |
| Germany | 2 (1.4%) | n/a |
| Italy | 7 (5.0%) | n/a |
| Japan | 2 (1.4%) | n/a |
| Lithuania | 1 (0.7%) | 1 (1.3%) |
| Netherlands | 11 (7.9%) | 10 (13.1%) |
| New Zealand | 3 (2.1%) | 1 (1.3%) |
| Poland | n/a | 1 (1.3%) |
| South Korea | 3 (2.1%) | n/a |
| Spain | 4 (2.87%) | 1 (1.3%) |
| Switzerland | 14 (10.07%) | 9 (11.8%) |
| Thailand | 1 (0.7%) | n/a |
| Turkiye | 3 (2.1%) | n/a |
| UK | 31 (22.3%) | 15 (19.7%) |
| USA | 55 (39.5%) | 36 (47.3%) |

***** The European Journal of Allergy and Clinical Immunology is based on several European countries.

Abbreviations: n/a, not available- the country had no representation in the specific category; RCT, randomised controlled trials; RW, real world studies.

Table S2. Total number of biologic agents used in the included studies in each analysis by year

| Publication year | | Dupilumab | Mepolizumab | Omalizumab | Mixed agents | Benralizumab | Reslizumab | Tezepelumab | Depemokimab | Total |
| --- | --- | --- | --- | --- | --- | --- | --- | --- | --- | --- |
|  | All studies 2006-2023 (Analysis A), N= 139 | | | | | | | | | |
| 2006 | | 0 | 0 | 0 | 0 | 0 | 1 | 0 | 0 | 1 |
| 2008 | | 0 | 0 | 1 | 0 | 0 | 0 | 0 | 0 | 1 |
| 2009 | | 0 | 0 | 1 | 0 | 0 | 0 | 0 | 0 | 1 |
| 2010 | | 0 | 0 | 1 | 0 | 0 | 0 | 0 | 0 | 1 |
| 2011 | | 0 | 1 | 0 | 0 | 0 | 1 | 0 | 0 | 2 |
| 2013 | | 0 | 0 | 3 | 0 | 0 | 0 | 0 | 0 | 3 |
| 2016 | | 1 | 0 | 0 | 0 | 0 | 0 | 0 | 0 | 1 |
| 2017 | |  | 2 | 0 | 0 | 0 | 0 | 0 | 0 | 2 |
| 2018 | | 1 | 0 | 1 | 0 | 0 | 0 | 0 | 0 | 2 |
| 2019 | | 3 | 2 | 2 | 0 | 1 | 0 | 0 | 0 | 8 |
| 2020 | | 7 | 3 | 8 | 1 | 4 | 0 | 0 | 0 | 23 |
| 2021 | | 12 | 4 | 2 | 4 | 7 | 0 | 0 | 0 | 29 |
| 2022 | | 14 | 5 | 3 | 7 | 5 | 0 | 0 | 0 | 34 |
| 2023 | | 13 | 4 | 3 | 9 | 1 | 0 | 1 | 0 | 31 |
| Total | | 51 | 21 | 25 | 21 | 18 | 2 | 1 | 0 | 139 |
|  | RCT and RW Studies 2006-2025 (Analysis B), N=76 | | | | | | | | | |
| 2006 | | 0 | 0 | 0 | 0 | 0 | 1 | 0 | 0 | 1 |
| 2010 | | 0 | 0 | 1 | 0 | 0 | 0 | 0 | 0 | 1 |
| 2011 | | 0 | 1 | 0 | 0 | 0 | 1 | 0 | 0 | 2 |
| 2013 | | 0 | 0 | 1 | 0 | 0 | 0 | 0 | 0 | 1 |
| 2016 | | 1 | 0 | 0 | 0 | 0 | 0 | 0 | 0 | 1 |
| 2017 | | 0 | 2 | 0 | 0 | 0 | 0 | 0 | 0 | 2 |
| 2018 | | 1 | 0 | 0 | 0 | 0 | 0 | 0 | 0 | 1 |
| 2019 | | 3 | 0 | 1 | 0 | 0 | 0 | 0 | 0 | 4 |
| 2020 | | 3 | 0 | 3 | 0 | 1 | 0 | 0 | 0 | 7 |
| 2021 | | 4 | 1 | 0 | 1 | 4 | 0 | 0 | 0 | 10 |
| 2022 | | 3 | 1 | 2 | 0 | 1 | 0 | 0 | 0 | 7 |
| 2023 | | 0 | 3 | 1 | 2 | 0 | 0 | 1 | 0 | 7 |
| 2024 | | 9 | 3 | 1 | 0 | 3 | 0 | 0 | 0 | 16 |
| 2025 | | 6 | 3 | 2 | 3 | 0 | 0 | 1 | 1 | 16 |
| Total | | 30 | 14 | 12 | 6 | 9 | 2 | 2 | 1 | 76 |

Table S3. The association between industry funding and the type of biologics

| **Predictor** | **Estimate** | **SE** | **Z** score | **P** **value** | **Odds ratio** | **95% Confidence Interval**  **[Lower-upper]** |
| --- | --- | --- | --- | --- | --- | --- |
| **Analysis A (2006-2023)** | | | | | | |
| Intercept | -3.00 | 1.02 | -2.92354 | 0.003 | 0.0500 | 0.00671- 0.373 |
| Dupilumab – Mixed | 2.56 | 1.06 | 2.40347 | 0.016 | 12.9032 | 1.60308- 103.858 |
| Mepolizumab – Mixed | 3.09 | 1.11 | 2.77482 | 0.006 | 22.0000 | 2.47866- 195.267 |
| Benralizumab – Mixed | 3.00 | 1.13 | 2.65596 | 0.008 | 20.0000 | 2.19248- 182.442 |
| Reslizumab – Mixed | 19.56 | 1696.73 | 0.01153 | 0.991 | 3.13e+8 | 0.00000- Inf |
| Omalizumab – Mixed | 2.59 | 1.10 | 2.34833 | 0.019 | 13.3333 | 1.53476- 115.834 |
| Tezepelumab - Mixed | 19.56 | 2399.54 | 0.00815 | 0.993 | 3.13e+8 | 0.00000- Inf |
| **Analysis B (2006-2025)** | | | | | | |
| Intercept | -1.61 | 1.10 | -1.46921 | 0.142 | 0.200 | 0.0234- 1.71 |
| Dupilumab – Mixed | 2.46 | 1.17 | 2.10762 | 0.035 | 11.667 | 1.1878- 114.59 |
| Mepolizumab – Mixed | 2.91 | 1.27 | 2.28232 | 0.022 | 18.333 | 1.5081- 222.88 |
| Benralizumab – Mixed | 2.30 | 1.30 | 1.76600 | 0.077 | 10.000 | 0.7765- 128.77 |
| Reslizumab – Mixed | 19.18 | 2797.44 | 0.00685 | 0.995 | 2.13e+8 | 0.0000- Inf |
| Omalizumab – Mixed | 1.27 | 1.24 | 1.02483 | 0.305 | 3.571 | 0.3130- 40.75 |
| Tezepelumab - Mixed | 19.18 | 2797.44 | 0.00685 | 0.995 | 2.13e+8 | 0.0000- Inf |
| Depemokimab – Mixed | 19.18 | 3956.18 | 0.00485 | 0.996 | 2.13e+8 | 0.0000- Inf |

* Note. Estimates represent the log odds of "Funding From Pharmaceutical Company = Yes" vs. "Funding From Pharmaceutical Company = No"

Abbreviations: HDI, human development index; SE, standard error;
